# Sex‐ and time‐specific parental effects of warming on reproduction and offspring quality in a coral reef fish

**DOI:** 10.1111/eva.13187

**Published:** 2021-01-13

**Authors:** Rachel K. Spinks, Lucrezia C. Bonzi, Timothy Ravasi, Philip L. Munday, Jennifer M. Donelson

**Affiliations:** ^1^ ARC Centre of Excellence for Coral Reef Studies James Cook University Townsville Queensland Australia; ^2^ Red Sea Research Center Division of Biological and Environmental Sciences and Engineering King Abdullah University of Science and Technology Thuwal Saudi Arabia; ^3^ Marine Climate Change Unit Okinawa Institute of Science and Technology Graduate University Kunigami‐gun Japan

**Keywords:** climate change, developmental plasticity, maternal effects, paternal effects, phenotypic plasticity, timing of exposure

## Abstract

Global warming can disrupt reproduction or lead to fewer and poorer quality offspring, owing to the thermally sensitive nature of reproductive physiology. However, phenotypic plasticity may enable some animals to adjust the thermal sensitivity of reproduction to maintain performance in warmer conditions. Whether elevated temperature affects reproduction may depend on the timing of exposure to warming and the sex of the parent exposed. We exposed male and female coral reef damselfish (*Acanthochromis polyacanthus*) during development, reproduction or both life stages to an elevated temperature (+1.5°C) consistent with projected ocean warming and measured reproductive output and newly hatched offspring performance relative to pairs reared in a present‐day control temperature. We found female development in elevated temperature increased the probability of breeding, but reproduction ceased if warming continued to the reproductive stage, irrespective of the male's developmental experience. Females that developed in warmer conditions, but reproduced in control conditions, also produced larger eggs and hatchlings with greater yolk reserves. By contrast, male development or pairs reproducing in higher temperature produced fewer and poorer quality offspring. Such changes may be due to alterations in sex hormones or an endocrine stress response. In nature, this could mean female fish developing during a marine heatwave may have enhanced reproduction and produce higher quality offspring compared with females developing in a year of usual thermal conditions. However, male development during a heatwave would likely result in reduced reproductive output. Furthermore, the lack of reproduction from an average increase in temperature could lead to population decline. Our results demonstrate how the timing of exposure differentially influences females and males and how this translates to effects on reproduction and population sustainability in a warming world.

## 1 INTRODUCTION

Reproduction is fundamental to sustaining viable populations. Reproductive activities generally occur within a narrow subset of the organism's entire thermal range, due to the energetic costs and physiological optimization that reproduction requires (Pörtner et al., 2006; Van Der Kraak & Pankhurst, 1997; Visser, 2008). Consequently, any changes in environmental temperature, such as human‐induced warming, can disrupt reproduction or influence the quantity and quality of offspring produced (Adams, 2010; Bokhorst et al., 2011; Pankhurst & Munday, 2011). To compensate for environmental temperature change, some organisms shift their location and/or reproductive phenology so that reproduction still occurs within the thermal optima (Ling et al., 2008; Poloczanska et al., 2013). However, these changes may result in a mismatch between reproduction and food availability for offspring when trophic levels are not similarly affected by temperature change (Visser & Both, 2005). Additionally, some species will be unable to shift timing or location to maintain reproduction at optimal temperatures and instead could adjust the thermal sensitivity of reproduction through processes such as phenotypic plasticity (nongenetic effects) and/or genetic evolution (Donelson et al., 2019). If shifts in reproductive timing or location, and/or adjustments to the thermal sensitivity of reproduction are not possible, there are likely to be serious consequences for population sustainability (Visser, 2008).

Due to the rapid rate of warming projected to occur over the coming decades, phenotypic plasticity is expected to be a critical mechanism by which organisms maintain performance in warmer conditions (Hendry et al., 2008; Munday et al., 2013). Phenotypic plasticity allows a genotype to produce an array of phenotypes under different environments (Stearns, 1989) and can be beneficial (i.e., adaptive) or maladaptive (Ghalambor et al., 2007). Whether phenotypic plasticity occurs may depend on the timing of exposure with early periods in development most sensitive to environmental change (West‐Eberhard, 2003). Environmental conditions experienced during early development can induce strong and permanent phenotypic change (i.e., developmental plasticity), whereas adult phenotypic adjustments are usually reversible (i.e., reversible plasticity) and are expected to be comparatively less sensitive (Angilletta, 2009).

There is evidence that phenotypic plasticity can mediate the effects of rising temperature on traits such as aerobic physiology, growth or behaviour (Forster et al., 2012; Nagelkerken & Munday, 2016; Seebacher et al., 2015); however, this means little if organisms cannot reproduce. For example, mosquitofish readily adjusted swimming speed to increased temperatures, yet will likely struggle to reproduce as sperm ceased to function at those same high temperatures (Adriaenssens et al., 2012; Wilson, 2005). Current knowledge about the effects of warming on reproduction and the potential for plasticity comes largely from research testing the potential for reversible plasticity on reproductive adults (e.g., Donelson et al., 2010; Fischer, Brakefield, et al., 2003; Miller et al., 2015; Suckling et al., 2015; Vilchis et al., 2005). When warming has instead occurred outside of this reproductive or postmaturity period, researchers generally exposed animals to increased temperatures for their entire life, making it impossible to disentangle the effects of temperature in development versus reproduction (for exceptions see Donelson et al., 2016; Fischer, Eenhoorn, et al., 2003; Fuxjäger et al., 2019; Huey et al., 1995; Stillwell & Fox, 2005). High temperature exposure at different life stages is especially relevant to heatwaves, which coincide with summer reproductive and early developmental windows for many organisms. Heatwaves are predicted to increase in frequency, intensity and duration due to global warming (Frölicher et al., 2018; Perkins‐Kirkpatrick & Gibson, 2017). To accurately predict responses of organisms to climate change, we require a greater understanding of how warming impacts reproduction depending on the timing of exposure and the capacity for adjustment through phenotypic plasticity.

While both parents contribute to offspring phenotype, mothers are generally expected to be more important due to their ability to make nongenetic contributions via provisioning or the transfer of mitochondria (Ho & Burggren, 2010; Mousseau & Fox, 1998). However, this classic idea is often shown to be a simplistic view of maternal and paternal contributions with both parents having both a genetic (i.e., DNA) and nongenetic/epigenetic influence (e.g., methylation, noncoding RNA or chromatin structure; Bonduriansky & Day, 2009). Furthermore, dependent on the reproductive strategy, sexes may have different capacity to adjust phenotypes, such as when only one parent provides parental care (Hunt & Simmons, 2000; Roth et al., 2012). For example, male stickleback fish solely care for eggs and juveniles and as such early offspring size was largely driven by paternal lifetime temperatures (Shama & Wegner, 2014; van Iersel, 1953). Whether environmental temperature experienced by parents will affect the phenotype of offspring can depend on the timing of thermal change, length of exposure and whether both parents experience the same thermal conditions (Donelson et al., 2018). Stillwell and Fox (2005) showed hatching success in a seed beetle is dependent on the interaction of the female's developmental and oviposition temperature, yet like many other studies the effect of warming to males is unknown. It is imperative we understand how the timing of exposure to both females and males affects reproduction and offspring performance if we are to predict the effects of warming on future population success.

Fishes are ectotherms with limited capacity for internal temperature regulation and, consequently, cellular function and physiological performance, including reproduction, are tightly linked to environmental temperature (Van Der Kraak & Pankhurst, 1997). Reproduction and embryogenesis are also the most thermally sensitive time for fishes (Dahlke et al., 2020). Temperature can directly affect fish reproduction ﻿by promoting or inhibiting hormone synthesis, altering hormone structure and modifying the action of hormones and enzymes in the hypothalamus, the pituitary and the gonads, resulting in changes to gamete and offspring quantity and quality (Pankhurst & Munday, 2011). For coral reef fishes, reproduction typically occurs during spring and summer. The repercussions of a 0.5–3°C increase in average summer temperature in coral reef fishes includes reduced or disrupted breeding, limited sperm production, and fewer and smaller offspring (Donelson et al., 2010; Kokita, 2003; Miller et al., 2015). However, most of these studies test adult fish for one breeding season under elevated temperatures and thus may not capture the full potential of thermal plasticity. When the full potential for thermal plasticity was explored, exposure to elevated conditions (+1.5°C) throughout development resulted in improved reproduction and offspring performance in some traits (i.e., beneficial developmental plasticity; Donelson et al., 2014). In addition, thermal conditions during reproduction can interact with those experienced during development to affect reproduction and offspring performance (Donelson et al., 2016) with some offspring traits, for instance sex ratio, only affected by the parent's developmental temperature (Donelson & Munday, 2015). A critical aspect of understanding the effects of environmental temperature change yet to be explored is whether timing of exposure differentially affects mothers and fathers and how this influences reproduction and newly hatched offspring.

The present study explores how the timing (developmental vs. reproductive) of exposure to simulated ocean warming affects reproduction and newly hatched offspring performance, and whether warming differentially affects mothers and fathers. For this study, we used the common coral reef damselfish, *Acanthochromis polyacanthus*, which forms monogamous pairs and provides biparental care. Specifically, male and female damselfish were reared from hatching in either a present‐day temperature (control) or an elevated temperature (+1.5°C). Once mature (1.5 years), fish were subsequently divided orthogonally into control and elevated reproductive temperatures to create pairs such that every thermal combination of sex and time occurred (eight pair combinations). A broad range of reproductive and hatchling traits were measured. Our experimental design tracked male and female family origins to estimate their contributions and separate plasticity (i.e., nongenetic effects) from family‐level effects. We hypothesized that parental developmental exposure to elevated temperature would benefit reproductive and hatchling traits, but reproduction in elevated temperature alone would result in negative effects. This is because *A*. *polyacanthus* appears to have limited capacity to adjust to warming as an adult in comparison with during development (Donelson et al., 2010, 2011; Rodgers et al., 2018; Spinks et al., 2019). Lastly, we expected female developmental exposure to higher temperature would have the greatest influence on reproductive traits, because of her larger initial investment (i.e., eggs), but both sexes would have a similar influence on hatchling traits since this species exhibits joint parental care.

## 2 METHODS

### 2.1 Experimental design

In the present study, we used the spiny chromis damselfish, *A. polyacanthus* (Bleeker 1855), which is common on coral reefs in the Indo‐Australian archipelago. Adult *A*.* polyacanthus* form monogamous pairs and breed primarily during the summer months (Robertson, 1973). Egg clutches adhere to the substrate with joint parental care and direct development taking place (Kavanagh, 2000; Pankhurst et al., 1999). Adult fish (F0 generation) were collected from the Palm Islands region (18°37′ S, 146°30′ E) of the central Great Barrier Reef in 2014 and 2015. Fish were transported to the Marine and Aquaculture Research Facility at James Cook University, Townsville, Australia, and housed in breeding pairs within 60 L aquaria, each with half a terracotta pot as a spawn site. Pairs were kept at seasonally cycling, present‐day temperatures approximating the Palm Islands region (AIMS, 2016). In the Austral summer of 2016, breeding bouts from six wild‐caught pairs were used in this experiment. Egg clutches were kept with the parents until hatching, allowing them to provide nest care as occurs in the wild.

**FIGURE 1:**
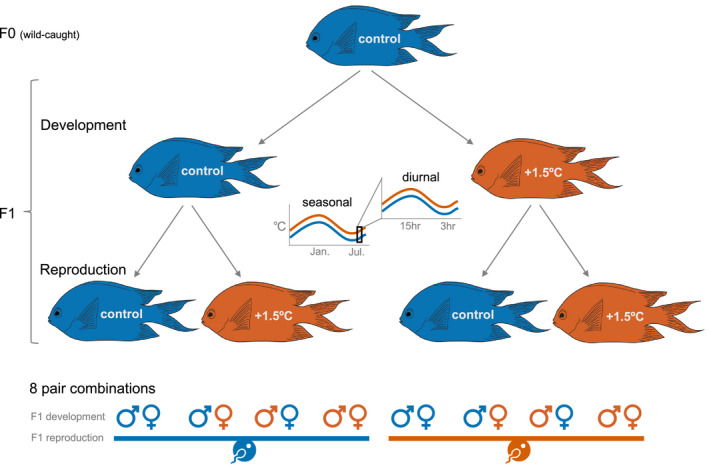
Experimental design. The F1 developmental split occurred shortly after hatching and the F1 reproductive split occurred around 1.5 years. Blue represents the present‐day control temperature (in summer 28.5°C with ±0.6°C diurnal variation), orange represents a temperature increase of 1.5°C (in summer 30.0°C with ±0.6°C diurnal variation)

The F1 generation were maintained in a 25,000 L recirculating system supplied with a continuous flow of natural seawater with precise temperature control. The system was divided into six blocks, each with its own sump, independent temperature control and approximately 40 42 L opaque tanks (6 × 2 kW Control Distributions custom‐built heaters; 18 kW Solarwise chiller EXC341RC). Water and air temperature were monitored continuously from a centralized environmental control system (PR Electronics temperature transmitter 5333A, ±0.1°C; Innotech Genesis II controller V5) and manually verified daily with a digital thermometer (±0.1°C, C26; Comark Instruments). Salinity, pH and nitrates were measured fortnightly and maintained around 35 ppm, 8.1 and below 20 mg/L, respectively. Water quality was maintained with mechanical, biological and ultraviolet filtration, protein skimming and partial water changes. An elevated temperature of +1.5°C was selected to match sea surface temperatures projected to occur on the Great Barrier Reef by 2050–2100 (Collins et al., 2013; Meehl et al., 2007) and to allow comparison with previous research on reproduction in similar populations (Donelson et al., 2010, 2014, 2016). This realistic average temperature increase already occurs during marine heatwaves (Frölicher et al., 2018; Spinks et al., 2019). The control water temperature simulated seasonal (winter minimum 23.2°C, summer maximum 28.5°C) and diurnal (03:00 hours −0.6°C, 15:00 hours +0.6°C) cycles for the Palm Islands region based on temperature loggers from 2002 to 2015 at 0.2–14.6 m depth (AIMS, 2016), with the elevated treatment matching this but 1.5°C higher (Figure 1). Similarly, the photoperiod of the Palm Islands region was replicated, reaching a maximum of 13 h 15 min light in summer (December) and a minimum of 11 h 01 min light in winter (June). Seasonal changes to water temperature and illumination were adjusted weekly.

In the Austral summer of 2016, newly hatched siblings (F1 generation) were split to be reared in a present‐day control temperature or +1.5°C (Figure 1). For each of the six families, fish were randomly allocated within 6 h of hatching to a minimum of five replicate tanks at each temperature, with approximately 10 fish per tank. Fish were given 2–3 h to slowly equilibrate to their rearing temperature via a 2 L tub floated in the tank and receiving a gradual inflow. Fish were fed live *Artemia nauplii* upon hatching and then weaned to commercial pellets (Appendix S1). At approximately 8 months of age, fish were sexed via external examination of the urogenital papilla (Hilder & Pankhurst, 2003) and permanently marked with colour elastomer tags (Northwest Marine Technology) to track developmental temperature, sex and family without further disturbance. By 1 year of age, fish were placed in sibling pairs to reduce competitive fighting. In the late Austral winter of 2017, when fish were approximately 1.5 years of age (i.e., maturation), all groups were adjusted to 24.5°C (±0.6°C diurnal variation) over a period of 1 week. This was to create nonsibling breeding pairs in preparation for the Austral summer breeding season of 2017/2018 (when fish were ~2 years old). The 24.5°C pairing temperature was a 1.3°C increase and a 0.2°C decrease from minimum winter temperature in the control and elevated temperature treatments, respectively. The breeding design included reciprocal sex crosses of the developmental temperatures resulting in four pair combinations of males and females reared in present‐day control and elevated temperatures, following Figure 1a in Bonduriansky et al. (2012). The four pair combinations were further divided into present‐day control and +1.5°C reproductive temperatures, which resulted in eight pair combinations (Figure 1). The eight pair combinations were replicated at least 20 times across three family crosses (family A × C, family B × D, family E × F) from the original six F0 families (see Figure 1a Bonduriansky et al., 2012). After 4 weeks of pairing, we gradually adjusted the fish to early spring temperatures over 2 weeks and re‐established the 1.5°C difference so that the control reproductive pairs were at 25.5°C ± 0.6°C and the elevated reproductive pairs were at 27°C ± 0.6°C by late September. Pairs were provided half a terracotta pot as a spawn site.

### 2.2 Reproduction and offspring traits

Summer temperatures were reached on the 8th of November 2017 and maintained until the last clutch hatched in May 2018 (28.5°C with ±0.6°C diurnal variation for control and 30.0°C with ±0.6°C diurnal variation for elevated reproductive temperatures). Tanks were checked daily for the presence of eggs. We calculated the probability of breeding during summer temperatures from pairs that had a minimum of 6 weeks together as this would indicate a stable pairing. When an egg clutch was discovered, an underwater photograph was taken (Canon G16 camera & housing) to determine the number of eggs laid. Clutch size for each pair was calculated from the first egg clutch photographed once summer temperature was reached, hereafter referred to as the first clutch. In 11 cases, pairs laid a clutch prior to the onset of summer temperatures. The total eggs per pair laid were summed from a maximum of two clutches. We could not calculate beyond two clutches as several pairs were sacrificed at this point for molecular research. Also, *A*.* polyacanthus* typically lay just 1–2 clutches per year in the wild (Thresher, 1985), so these first two clutches are ecologically relevant. For the total eggs laid calculation, we only included pairs that stayed together at least 6 weeks since their first clutch hatched. Once the first clutch was photographed, 10 eggs were sampled from random locations within the clutch and photographed to determine egg area (±0.01 mm^2^). Clutches were kept with the parents allowing them to provide nest care as occurs in the wild. On day eight, the first clutch was photographed again to determine embryonic mortality. Eggs no longer present (most likely removed by parents) or that had not developed were considered deceased. Embryonic duration was estimated from the first clutch beginning the day it was laid until it hatched. Within hours of hatching, 20 offspring from each clutch were euthanized by an overdose of clove oil. They were weighed (±0.1 mg; excess water removed with a Kimwipe) and then preserved in phosphate‐buffered formaldehyde (4%) to photograph within 48 h to determine hatch standard length (±0.01 mm) and hatch yolk area (±0.01 mm^2^). We were unable to measure the standard length of four hatchlings or yolk area of seven hatchlings due to mishandlings after weighing. Clutch size at laying and day eight, egg area, hatch standard length and hatch yolk area were measured blind by the same person (B. Spady) using ImageJ software v. 1.50i (Schneider et al., 2012). This research was conducted under James Cook University's animal ethics approval A1990, A2210 and A2315.

### 2.3 Statistical analyses

We used the rstanarm package v.2.18.2 (Goodrich et al., 2020) to implement Bayesian mixed models. We tested whether reproductive and offspring performance for the pairs with various sex and life‐stage exposures to warming differed compared with pairs exposed their entire lives to present‐day control temperature. The F1 thermal experience (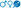, 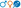, 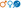, 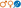, 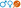, 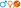, 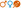, 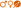) was an independent variable in all models, with the control group set as the intercept (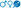). The intercept varied by male family (A‐F) and female family (A‐F) so that the variance attributed to paternal and maternal family‐level effects could be estimated. Additionally, the intercept varied by pair replicate, defined as pairs from the same family cross within treatments, to prevent pseudoreplication (Arnqvist, 2019). For the dependent variables: egg area, embryonic mortality, hatch weight, standard length and yolk area, where multiple eggs or hatchlings came from a pair within a group of pairs from the same family cross and treatment, the intercept varied by pair nested in pair replicate. This was in addition to male and female family. Again, this ‘random’ effect structure prevented pseudoreplication (Arnqvist, 2019) and accounted for the hierarchical nature of the experimental design. We further explored whether both slopes and intercepts varied (i.e., random‐slope random‐intercept model) in egg area, embryonic mortality, hatch weight, standard length and yolk area since they had a larger sample size. Based on visual inspection and Bayesian leave‐one‐out information criterion (LOOIC; Vehtari et al., 2017), hatch weight and yolk area slopes differed between the dependent variable and the F1 treatments (i.e., a full random‐slope random‐intercept model fitted best). Variances attributed to ‘random’ effects are always stated in the model link scale. See Appendix S1 for model distributions and links.

Mother size was initially considered a covariate for the dependent variables clutch size, total eggs per pair and egg area because they often correlate (Lim et al., 2014). However, we found no clear correlations and model fits visually and via LOOIC improved when excluding mother size (Appendix S2). This may be due to mother size measurements being taken at different time points (most females were measured at the end of the breeding season to prevent disturbance, but some were measure when euthanized for molecular research), or because of limited size differences as all fish were the same age. The general conclusions were the same with and without mother size and no interactions were present so we selected the most parsimonious models (Appendix S2).

Bayesian models allow integration of prior knowledge (Kruschke, 2015). We specified weakly informative priors using rstanarm except when a more informative prior was required to allow regularization, or because specific knowledge existed (Table S1 of Appendix S1; Donelson et al., 2010, 2014, 2016). The posterior distribution is derived from the prior distribution (previous evidence) and the likelihood function (new evidence). Visual posterior checks confirmed that priors never heavily influenced the posterior. Using the Hamiltonian Monte Carlo algorithm, models were run with three chains by means of the No‐U‐Turn sampler for a minimum of 5000 iterations with every second or third posterior sample thinned and the first 10–50% discarded depending on the complexity of the model (Appendix S2). Model validation and selection followed Spinks et al. (2019). The probability that a treatment was smaller or larger relative to the control group is calculated from the posterior distribution (Appendix S2). Probabilities are expressed as a per cent and the closer they are to 100% suggests greater confidence in a treatment being smaller or larger relative to the control group, whereas nearer to 50% suggests little confidence in a treatment being smaller or larger relative to the control group. Highest posterior density credible intervals (analogous to Frequentist confidence intervals) are used in all figures. Analyses were performed in R v.3.6.0 (R Core Team, 2020) with figures created in the ggplot2 package v.3.1.1 (Wickham, 2016).

## 3 RESULTS

**FIGURE 2:**
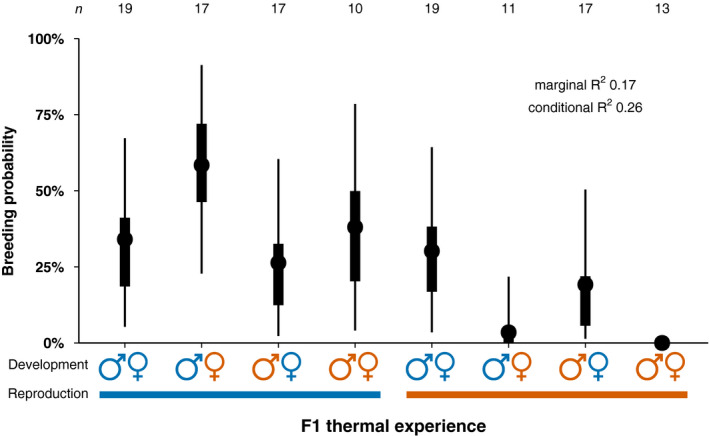
Bayesian posterior median values (circles), 50% credible intervals (rectangles) and 95% credible intervals (thin lines) of the breeding probability, *n* = pairs. Blue represents the present‐day control temperature while orange represents a temperature increase of 1.5°C

Pairs comprised of a male and female that developed and reproduced at control temperature (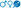) had a 34% median breeding probability (Figure 2; Table S2 of Appendix S1). Whereas no pairs bred when both males and females were exposed to elevated temperature during their developmental and reproductive stages (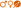), resulting in a 99.98% probability of fewer breeders compared with pairs exposed their entire lives to control temperature (Figure 2; Table S2 of Appendix S1). Similarly, pairs only bred once when males were exposed to control developmental temperature, females exposed to higher developmental temperature, and reproduction occurred at higher temperature (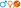; 31% median decrease in breeding probability), with a 98% probability of fewer breeders compared with pairs exposed their entire lives to control temperature (Figure 2; Table S2 of Appendix S1). By contrast, there was a 24% median increase in breeding probability for pairs where males developed at control, females developed at high temperature, and reproduction occurred at control temperature (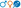), resulting in a 85% probability of more breeders than when females also developed at control temperature (Figure 2; Table S2 of Appendix S1). In all other treatments (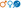, 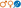, 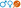, 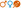), the breeding probability was similar to that of pairs where male, female and reproduction were in control temperature (Figure 2; Table S2 of Appendix S1). The variance observed in breeding probability was partly due to some pair replicates breeding and others not (‘random’ effect pair replicate *σ* 1.20 log odds); however, this was generally smaller than the magnitude of treatment effects (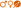 ‐17.87, 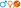 ‐2.86, 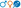 ‐1.03 log odds). Family effects contributed the least variance to breeding probability with the among male family standard deviation (0.57 log odds) less than female family (0.68 log odds). Further analyses of reproductive and hatchling traits exclude the treatment where males developed at control temperature, females developed at elevated temperature, and reproduction occurred at elevated temperature (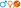) because of the uncertainty around a sample size of one and the exceptionally high embryonic mortality (74%) experienced by this clutch (Appendix S2).

**FIGURE 3:**
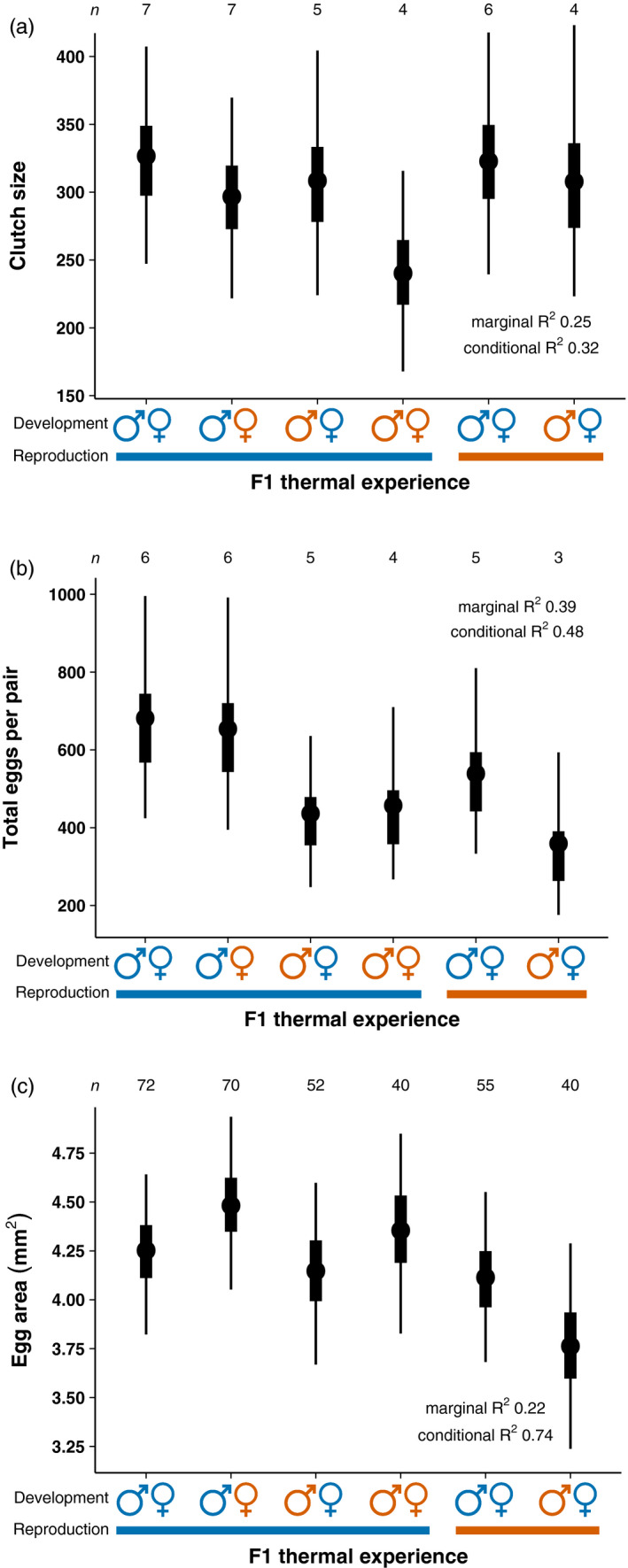
Bayesian posterior median values (circles), 50% credible intervals (rectangles) and 95% credible intervals (thin lines) of (a) clutch size, *n* = pairs, (b) total eggs per pair, *n* = pairs (c) egg area, *n* = eggs. Blue represents the present‐day control temperature while orange represents a temperature increase of 1.5°C

Clutch size and total eggs laid per pair decreased when fathers or both parents were exposed to higher temperature during development, or when pairs were exposed to higher temperature during reproduction. Clutch size was similar across treatments except for pairs where both sexes developed at increased temperature but reproduced at control temperature (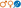; Figure 3a; Table S2 of Appendix S1). These pairs had a 96% probability of producing smaller clutches and a median of 87 fewer eggs per clutch compared with pairs exposed their entire lives to control temperature (Figure 3a; Table S2 of Appendix S1). Family effects provided minimal variance to clutch size compared with the magnitude of the treatment effect (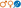 ‐0.31 log), with the among male family standard deviation (0.01 log) less than female family (0.02 log). Since pairs where both sexes developed in higher temperature but reproduced in control temperature (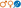) laid smaller clutches, it was not surprising that they also produced a median of 224 fewer eggs in total over the breeding season, resulting in a 94% probability of less eggs laid relative to pairs exposed their entire lives to control temperature (Figure 3b; Table S2 of Appendix S1). In contrast, three treatments (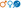, 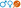, 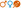) produced similar size clutches, but laid fewer total eggs per pair due to approximately half the pairs in these treatments producing only one clutch. A median of 245 fewer eggs in total were laid by pairs comprised of a male reared in elevated temperature, a female reared in control temperature and reproduction in control temperature (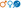), resulting in a 96% probability of less eggs laid relative to pairs exposed their entire lives to control temperature (Figure 3b; Table S2 of Appendix S1). When reproduction occurred at elevated temperature (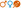), a median of 322 fewer eggs in total were laid and a 97% probability of less eggs produced compared with pairs exposed their entire lives to control temperature (Figure 3b; Table S2 of Appendix S1). For pairs where both sexes developed in control temperature but reproduction occurred at higher temperature (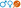), there was a median of 142 fewer eggs laid in total, resulting in a 84% probability of less eggs produced relative to pairs exposed their entire lives to control temperature (Figure 3b; Table S2 of Appendix S1). Finally, pairs comprised of males reared in control temperature, females reared in increased temperature and reproduction in control temperature (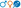) produced similar total number of eggs to that of pairs where males, females and reproduction were in control water temperature (Figure 3b; Table S2 of Appendix S1). Family effects provided minimal variance to the total number of eggs laid compared with the magnitude of treatment effects (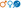 ‐0.45, 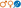 ‐0.41, 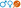
‐0.23, 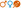 ‐0.63 log), with the among male family standard deviation (0.04 log) less than female family (0.06 log).

Egg area increased slightly when females developed in warmer waters, yet decreased if males developed and reproduced at higher temperature. Pairs comprised of males in control temperature, females in elevated temperature and reproduction in control temperature (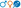) had a median increase in egg area of 0.23 mm^2^ and 83% probability of larger eggs relative to pairs exposed their entire lives to control temperature (Figure 3c; Table S2 of Appendix S1). Conversely, pairs comprised of males in warmer water, females in control temperature and reproduction in warmer water (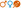) had a median decrease in egg area of 0.49 mm^2^ and 96% probability of smaller eggs relative to pairs exposed their entire lives to control temperature (Figure 3c; Table S2 of Appendix S1). Egg area in all other treatments (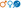, 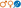, 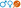) was similar to pairs exposed their entire lives to control temperature (Figure 3c; Table S2 of Appendix S1). The variance observed in egg area was moderately explained by the ‘random’ effects, that is male family, female family and pair nested in pair replicate (see marginal vs. conditional *R* ^2^ Figure 3c and Appendix S2). Specifically, the largest contributor of variance was pair (*σ* 0.14 mm^2^) although this was smaller than the magnitude of treatment effects, meaning egg area varied between pairs but we could still observe differences due to the F1 thermal experience. Conversely, family provided the least variance to egg area with the among male family standard deviation (0.03 mm^2^) slightly less than female family (0.05 mm^2^).

Embryonic duration depended on parental exposure, whereas embryonic mortality was mostly explained by among pair variation. Embryonic duration reduced from 9 days (control 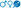) to 8 days when the parents' reproductive temperature was elevated, irrespective of the parents’ developmental environment (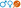 and 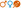; Figure S1A and Table S2 of Appendix S1). The probability of a shorter embryonic duration for offspring of 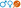 and 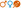 was 99.8% and 99% compared with parents exposed their entire lives to control temperature (Figure S1A and Table S2 of Appendix S1). Pairs comprised of males reared in higher temperature, females reared in control temperature and reproduction in control temperature (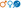) and pairs where females also developed in higher temperature (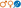) experienced an increase in their offspring's embryonic duration by half a day with a 89% and 96% probability of a longer embryonic duration relative to parents exposed their entire lives to control temperature (Figure S1A and Table S2 of Appendix S1). The embryonic duration of 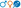 was similar to control pairs (Figure S1A and Table S2 of Appendix S1). Family provided the least variation to embryonic duration compared with the magnitude of treatment effects, with the among male family and female family standard deviation equalling 0.04 days. Conversely embryonic mortality, which ranged from 4 to 13% median mortality, was largely explained by the ‘random’ effects, that is male family, female family and pair nested in pair replicate (see marginal vs. conditional *R* ^2^ Figure S1B and Table S2 of Appendix S1 and Appendix S2). Specifically, the among‐pair standard deviation (4.85 log odds) was greater than the magnitude of the largest treatment effect (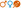 1.15 log odds), meaning that embryonic mortality varied substantially between pairs making it difficult to determine differences solely due to the F1 thermal experience.

**FIGURE 4:**
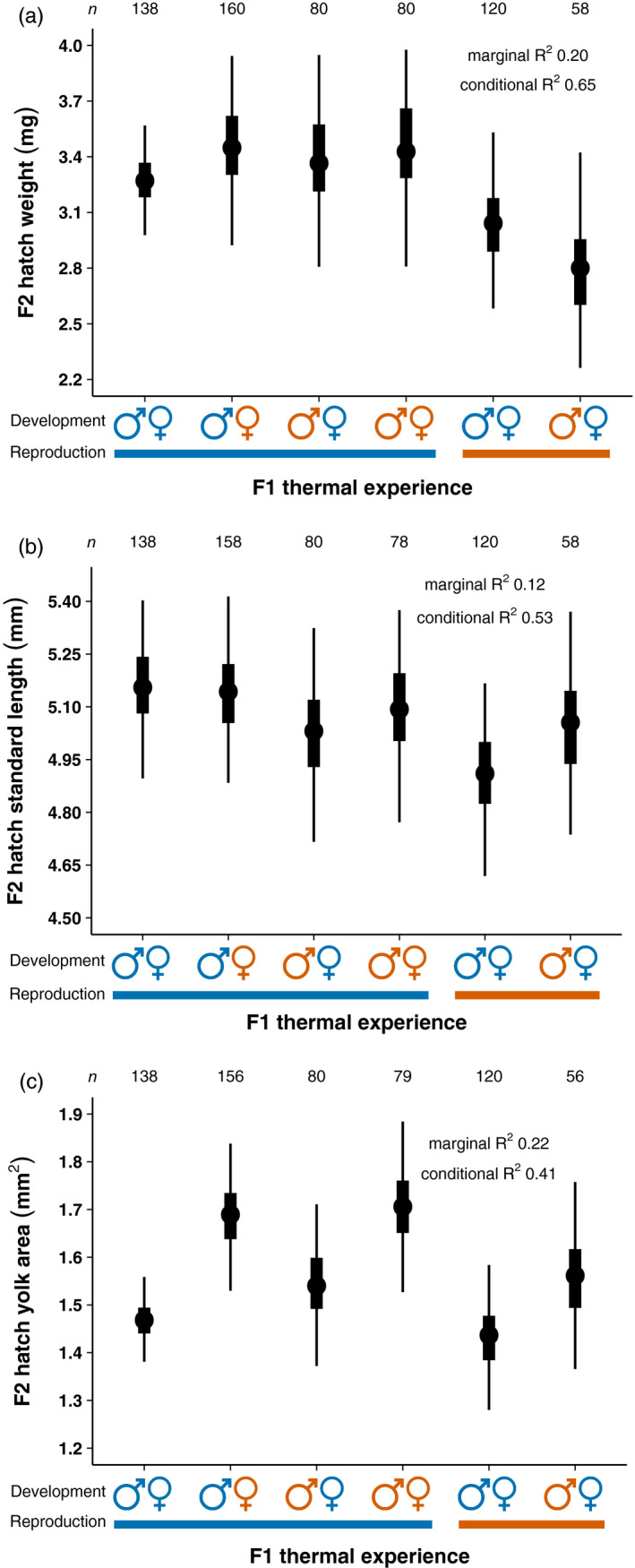
Bayesian posterior median values (circles), 50% credible intervals (rectangles) and 95% credible intervals (thin lines) of (a) weight, (b) standard length and (c) yolk area at hatching, *n* = hatchlings. Blue represents the present‐day control temperature while orange represents a temperature increase of 1.5°C

Weight at hatching decreased when parents were exposed to higher reproductive temperature. Pairs comprised of a male and female that developed in control temperature and reproduced in elevated temperature (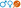) produced hatchlings that weighed a median of 0.2 mg less and a 82% probability of weighing less compared with offspring from parents exposed their entire lives to control temperature (Figure 4a; Table S2 of Appendix S1). Pairs where males also developed at elevated temperature (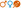) similarly produced offspring that weighed a median of 0.5 mg less with a 93% probability of weighing less compared with offspring from parents exposed their entire lives to control temperature (Figure 4a; Table S2 of Appendix S1). The rest of the treatments (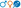, 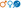, 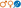) produced offspring similar in hatch weight to offspring from parents exposed their entire lives to control temperature (Figure 4a; Table S2 of Appendix S1). The variance observed in hatch weight was moderately explained by the ‘random’ effects, that is male family, female family and pair nested in pair replicate (see marginal vs. conditional *R* ^2^ Figure 4a and Appendix S2). Specifically, the largest contributor of variance was pair (*σ* 0.04 mg) although this was smaller than the magnitude of treatment effects, meaning hatch weight varied between pairs but we could still observe differences due to the F1 thermal experience. Family effects provided a similar amount of variance to hatch weight, with the among male family standard deviation (0.03 mg) much greater than female family (<0.001 mg).

Standard length at hatching decreased when parents were exposed to elevated reproductive temperature. Pairs comprised of a male and a female that developed in control temperature but reproduced in higher temperature (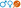) produced hatchlings that were a median of 0.24 mm shorter and a 97% probability of shorter length compared with offspring from parents exposed their entire lives to control temperature (Figure 4b; Table S2 of Appendix S1). While there was a small decrease in hatch standard length for 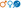 (−0.13 mm and 83% probability), this and the other treatments (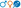, 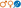, 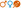) produced offspring similar in length to offspring from parents exposed their entire lives to control temperature (Figure 4b; Table S2 of Appendix S1). The variance observed in hatch standard length was moderately explained by the ‘random’ effects, that is male family, female family and pair nested in pair replicate (see marginal vs. conditional *R* ^2^ Figure 4b and Appendix S2). Specifically, the largest contributor of variance was male family and female family (*σ* 0.03 mm for each) although this was smaller than the magnitude of treatment effects, meaning hatch standard length varied between father families and between mother families, but we could still observe differences due to the F1 thermal experience.

Yolk area at hatching increased when mothers developed at higher temperature, irrespective of the father's developmental temperature. Pairs where males developed in control conditions, females developed in higher temperature, and reproduction occurred in control conditions (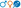) produced newly hatched offspring with a median of 0.22 mm^2^ more yolk and a 99.3% probability of increased yolk area compared with offspring from parents exposed their entire lives to control temperature (Figure 4c; Table S2 of Appendix S1). Pairs comprised of a male and a female reared in higher temperature and with reproduction occurring in control temperature (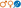) similarly produced newly hatched offspring with a median of 0.24 mm^2^ more yolk and a 99% probability of increased yolk area compared with offspring from parents exposed their entire lives to control temperature (Figure 4c; Table S2 of Appendix S1). While a slight increase in hatch yolk area for 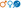 (0.07 mm^2^ and 79% probability) and 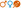 (0.09 mm^2^ and 83% probability) was observed, these and 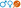 produced newly hatched offspring with yolks closer in size to that of parents exposed their entire lives to control temperature (Figure 4c; Table S2 of Appendix S1). The variance observed in hatch yolk area was moderately explained by the ‘random’ effects, that is male family, female family and pair nested in pair replicate (see marginal vs. conditional *R* ^2^ Figure 4c and Appendix S2). Specifically, the largest contributor of variance was pair (*σ* 0.005 mm^2^) although this was smaller than the magnitude of treatment effects, meaning hatch yolk area varied between pairs but we could still observe differences due to the F1 thermal experience. Family provided the least variation to hatch yolk area with the among male family and female family standard deviation equal (0.002 mm^2^). Summary statistics of all traits are found in Table S2 of Appendix S1.

## 4 DISCUSSION

Successful reproduction is vital to ensure the persistence of populations and species. Our results show that the timing (developmental or reproductive) of exposure to a 1.5°C increase in water temperature influenced fecundity and hatchling performance in a coral reef fish, and these impacts differed depending on the sex of the parent exposed. Specifically, developmental exposure to warming by females enhanced reproduction and offspring quality, whereas developmental exposure by males reduced reproductive output. When both sexes developed in warm water, we observed a combination of the effects for male and female development. Reproduction only, or the combination of developmental and reproductive exposure to elevated temperature by either or both sexes had negative consequences on reproductive output and offspring quality. While female development in warm water may improve reproductive performance at current‐day temperature, all other combinations of exposure to warming resulted in fewer and/or poorer quality offspring or disrupted reproduction, which could lead to population decline. Our results highlight the complexity of predicting the effects of ocean warming on a population since thermal effects to reproduction interact across life stages and sexes. They also show that some species may lack the ability for plasticity to maintain reproductive performance in a rapidly warming climate. In a climate change context, heatwaves could generate a mismatch in developmental temperatures by males or females from one cohort or year breeding with fish from another cohort or year that were not exposed to a heatwave. Heatwaves could also result in fish experiencing higher temperatures during development, but not at reproduction.

Developmental exposure of females to increased temperature enhanced some reproductive and offspring characteristics. When only females developed in warmer conditions, and reproduction occurred in control conditions, more pairs reproduced, larger eggs were laid, and hatchlings had larger yolks in comparison with pairs in control conditions. The obvious benefit being that more pairs breeding increases the total number of offspring produced, while progeny developing from large eggs or hatchlings with large yolks may grow faster, attain greater size, and are more likely to survive (Bagenal, 1969; Brooks et al., 1997; Fox, 1994; Meekan et al., 2006). Our results are in contrast to research where higher female developmental temperature resulted in smaller eggs in butterflies (Fischer, Eenhoorn, et al., 2003), lower lifetime fecundity in seed beetles (Stillwell & Fox, 2005) or lower mating success in stickleback fish (Fuxjäger et al., 2019). The reproductive changes observed in *A*.* polyacanthus* were likely the result of developmental plasticity of the female's endocrine system, perhaps shifting the thermal optimum for reproductive functioning. Changes to gene expression levels have previously been observed in female *A*.* polyacanthus* that developed at an elevated temperature (+3°C), with higher expression of the Cyp11b1 gene measured in the ovaries compared with fish reared at control temperature (Veilleux et al., 2018). The encoded protein of Cyp11b1 converts testosterone to the active metabolite 11‐ketotestosterone (11 KT), although mostly used by male fishes 11 KT has been shown to accelerate development of the ovaries in cod and eels (Borg, 1994; Kortner et al., 2009; Lokman et al., 2002; Sudo et al., 2012). Accordingly, female *A*.* polyacanthus* reared in warmer water may experience rapid development of their ovaries such that they are better prepared when reproduction occurs at control temperature for that first breeding season compared with females reared in control temperature.

Developmental exposure of males to increased temperature decreased reproductive output. When only males developed at higher temperature and then pairs reproduced at control conditions, fewer clutches were produced and embryonic durations increased. Interestingly, reduced expression of follicle‐stimulating hormone receptor (Fshr) and luteinizing hormone receptor (Lhcgr) genes were found in the testes of *A*.* polyacanthus* reared in +3°C relative to males reared in control temperature (Veilleux et al., 2018). These receptors are essential to bind the gonad‐stimulating hormones, and their reduced expression could play a role in the downturn in reproductive output. Lastly, when both sexes developed in warm water and then reproduced at control conditions, a combination of the effects observed for male and female developmental exposure alone occurred. Specifically, clutches were smaller resulting in fewer total eggs per pair, embryonic development increased and hatchlings had larger yolks compared with pairs in control conditions. Similarly, *A*.* polyacanthus* pairs developing at +3°C and reproducing at control conditions produced smaller clutches, fewer total eggs and larger yolks (Donelson et al., 2016). While larger eggs or yolks likely increase offspring growth and survival, they drain resources from mothers and this typically results in a trade‐off between offspring size/quality and the number of offspring produced (Fox et al., 1997; Lim et al., 2014). The pattern of producing clutches of fewer but higher quality offspring, as observed when mothers and fathers were exposed to warming in development, could be an adaptive strategy when larger offspring are disproportionately selected for in certain environmental conditions (Fox et al., 1997).

Reproductive exposure to warming resulted in fewer and poorer quality offspring. Specifically, when both sexes developed at control conditions but reproduced in warmer water, pairs produced fewer clutches, embryos developed faster, and hatchling weight and standard length decreased compared with pairs that reproduced in control conditions. Similarly, reproductive and offspring characteristics were negatively impacted when anemonefish, red abalone, butterflies and stickleback fish were exposed to elevated temperature only as adults (Fischer, Eenhoorn, et al., 2003; Fuxjäger et al., 2019; Miller et al., 2015; Vilchis et al., 2005). Reproduction in fish typically occurs within a narrow thermal window and our results suggest that an increase of +1.5°C would push summer temperature beyond the optimal window for reproduction in *A*.* polyacanthus*. This is consistent with an increase in exercise‐related mortality at 1.5°C above average summer temperatures in a low latitude population of *A*.* polyacanthus* (Rodgers et al., 2018), which suggests that *A*.* polyacanthus* populations already live close to their thermal optimum. Further, a shorter embryonic duration and smaller hatch size of offspring from parents that reproduced in warmer temperature are consistent with the effects of temperature on developmental and metabolic rates in fishes (Munday et al., 2008). Since the embryos developed in the same temperature as their parents and metabolic rates are known to increase with temperature, this leads to a faster embryonic development and concomitantly smaller size at hatching.

Developmental and reproductive exposure to warming by either or both sexes generally had negative synergistic effects on reproduction and offspring. Solely male development in higher temperature lead to a lower reproductive output, whereas pairs reproducing in higher temperature produced faster developing embryos and fewer and poorer quality hatchlings. When males were exposed to higher temperature in both developmental and reproductive life stages, but females developed in control conditions, we observed the same negative effects for development and reproduction alone, but they were generally larger in magnitude, plus egg size was also impacted. Thus, prolonged exposure to higher temperature by males would likely have substantial effects on reproductive output in a future warmer ocean. However, it should be noted that although males developing and reproducing in higher temperature produced fewer and poorer quality offspring, the pairs still bred in similar proportion to control pairs. Conversely, only one breeding pair reproduced when females had prolonged exposure to warming, irrespective of the males’ developmental temperature (0%–3% median breeding probability) and the single clutch they produced had very high embryonic mortality. Our findings reflect previous work on *A*.* polyacanthus* where life‐long increased temperatures for both sexes resulted in cessation of or a decline in breeding (Donelson et al., 2014, 2016), but our results suggest it is likely the effect of elevated temperature to females that is driving this response. Our results also demonstrate that female developmental exposure to warming does not necessarily allow developmental plasticity to maintain reproductive performance if warming continues past development. Similarly, female seed beetles exposed to higher temperature during development and reproduction had a lower lifetime fecundity than beetles exposed to higher temperature in only one life stage or not at all (Stillwell & Fox, 2005). The negative effects on reproduction in our study by prolonged exposure to warming for either sex could be explained by a chronic stress response, where the focus is switched to other physiological processes for survival at the expense of reproduction. Normally, the stress axis (hypothalamic–pituitary–interrenal axis in fish) manages change through the release of glucocorticoid hormones with the aim to maintain homeostasis (Beldade et al., 2017). Prolonged stress (i.e., warming) may cause persistently elevated glucocorticoid hormones, shifting the hormone baseline such that homeostatic overload occurs (Angelier & Wingfield, 2012; Pankhurst & Munday, 2011; Romero et al., 2009). This is demonstrated in the correlation between reduced fecundity and hormonal stress responses of wild anemonefish living on bleached anemones during a marine heatwave (Beldade et al., 2017). Overall, this implies the duration of exposure to increased temperature by males and females is important to consider, and that prolonged exposure to warming will likely result in population declines as a consequence of marked reductions in reproductive output.

While family had an influence on reproduction and offspring performance, the magnitude of the effect was smaller than parental exposure to warming. This confirms that the previously discussed thermal effects are indeed due to phenotypic variation in these traits. This might suggest there is limited ability for *A*.* polyacanthus* to genetically adapt to warming in terms of the reproductive and offspring traits we measured. However, we only had six families to start the experiment and could not instigate a diallel breeding design that would enable us to estimate additive genetic effects with confidence (Munday et al., 2013). Male and female family effects in our analysis are likely to reflect both genetic variance and some nongenetic effects. Nevertheless, family‐level effects were comparatively minor compared with the treatment effects, suggesting that the genetic variation in the reproductive traits measured is not especially high. More generally, genetic variance in fitness related traits is predicted to be low because strong selection on such traits will erode genetic variance through time (Fisher, 1930; Mcfarlane et al., 2014; Teplitsky et al., 2009). Indeed, Salles et al. (2020) recently demonstrated very low genetic variance in lifetime reproductive success in a wild clownfish population. By contrast, we have previously demonstrated there is substantial additive genetic variance in metabolic traits and growth rate in *A*.* polyacanthus*, including at +1.5°C (Munday et al., 2016). Although family‐level effects on reproduction were minimal, we found female family provided greater variation in breeding and clutch related traits than male family, which likely reflects some component of maternal effects in addition to genetic effects. Male family provided greater variation in hatching weight than females, while males and females contributed equally to family‐level variation in the remainder of the traits.

One striking difference between this present study and previous work is the addition of daily temperature cycles. By incorporating a diurnal temperature cycle of ±0.6°C, which mimicked natural conditions of the collection location of the wild‐caught generation, the effects of warming on reproduction and offspring performance appear to be accentuated. Previous findings suggest that *A*.* polyacanthus* from the same region of the Great Barrier Reef can restore their reproductive capacity to control levels with stable +1.5°C for one generation (Donelson et al., 2014). Yet, we observed disrupted breeding in pairs of males and females exposed during developmental and reproductive periods to +1.5°C with a daily variation, which instead matches previous results for *A*.* polyacanthus* reared at a stable +3°C (Donelson et al., 2010, 2014, 2016). This could mean more dramatic effects to reproduction and offspring performance will occur in natural settings at a lower increase than stable temperature experiments suggest. This is interesting since predictable environmental variability, like diurnal temperature variation, may be expected to promote adaptive plasticity but when organisms exist near their thermal limits, as coral reef fishes often do, it's not surprising that thermal variability exacerbates effects (Kroeker et al., 2020; McLeod et al., 2014; Rummer et al., 2014). Accordingly, this highlights the importance of replicating natural conditions as much as possible in experimental settings to accurately predict climate change impacts.

The thermal history of organisms can impact reproductive output and offspring quality. This study shows that the effects of ocean warming can be sex and exposure timing specific and additionally these effects occur in synergy, additively and in opposing directions, thus making the projection for a population response to future warming highly complex. Further, it suggests that while plasticity to warming may be adaptive for some organisms (e.g., Sandoval‐Castillo et al., 2020), it may not be for others. This study also stresses the importance of producing the most relevant simulations of environmental change feasible in the laboratory, as aspects like natural diurnal cycles may influence phenotypes. Our study highlights the importance of considering life‐stage and sex‐specific exposures to warming to accurately predict how populations and species may cope with climate change.

## CONFLICT OF INTEREST

The authors declare there are no conflicts of interest.

## Supporting information

Appendix S1

Appendix S2

## Data Availability

Data for this study are available at James Cook University's Tropical Data Hub https://doi.org/10.25903/5f14fa3fafaba, and the R script used in analyses is provided in the Supporting information.
