# Supplementary material for: Sex‐ and time‐specific parental effects of warming on reproduction and offspring quality in a coral reef fish
Source: Evol Appl. 2021 Jan 13;14(4):1145–58. doi: 10.1111/eva.13187 (PMC8061261; doi:10.1111/eva.13187)
Supplement: Supplementary file 1 — Appendix S1 [file EVA-14-1145-s001.docx]

**Appendix S1**

Spinks, R.K., Bonzi, L.C., Ravasi, T., Munday, P.L., and Donelson, J.M. (2021). Sex- and time-specific parental effects of warming on reproduction and offspring quality in a coral reef fish. *Evolutionary Applications*, DOI: 10.1111/eva.13187

**Feeding protocols**

Newly hatched *Acanthochromis polyacanthus* were fed live *Artemia nauplii* the first three days then weaned to 200–400 µm NRD pellets (INVE Aquaculture ﻿Salt Lake City, UT, USA) supplied daily at 5.18mg/fish and from 46 days post hatching (dph) at 21.7mg/fish. Between 96 dph and 300 dph 500–800 µm ﻿NRD pellets were supplied daily initially at 29.29 mg/fish and then increased to 58.56 mg/fish. After this period *A*. *polyacanthus* were given G12 adult breeder pellets at least once per day until satiation.

**Model distributions and links**

The dependent variables egg area, embryonic duration, hatch weight, hatch standard length, and hatch yolk area were modelled with a Gaussian distribution and an identity link (i.e. LMM). Embryonic duration is a count and therefore a Poisson distribution is normally expected, but due to underdispersion we found a Gaussian distribution with narrow priors provided the best fit. It was possible to use a Gaussian distribution here because no relationship existed between the mean and variance and means did not approach zero. The dependent variables clutch size and total eggs per pair being counts were modelled with a Poisson distribution and a log link (i.e. GLMM). To manage overdispersion in these Poisson models we included observation-level random effects, where each data point (i.e. pair) receives a unique level of a random effect (Harrison, 2014). We found observation-level random effects were a better solution visually and via Bayesian leave-one-out information criterion (LOOIC; Vehtari, Gelman, & Gabry, 2017) than using a negative Binomial distribution. Observation-level random effects were excluded in conditional R^2^ calculations as in this circumstance it has little biological meaning (Harrison, 2014). Lastly, the dependent variables breeding probability and embryonic mortality were modelled with a Binomial distribution and logit link (i.e. GLMM) due to their binary properties.

**Table S1.** Priors used in each model.

| Model | Intercept | Slope | Error standard deviation |
| --- | --- | --- | --- |
| Breeding probability | *Normal*(0, 20 log odds) | *Normal* (0, 20 log odds) | – |
| Clutch size | *Normal*(0, 10 log) | *Normal* (0, 2.5 log) | – |
| Total eggs per pair | *Normal*(0, 10 log) | *Normal* (0, 2.5 log) | – |
| Egg area | *Normal*(0, 5.12 mm^2^) | *Normal* (0, 1.28 mm^2^) | *Exponential* (rate 0.51) |
| Embryonic duration | *Normal*(10, 3 days) | *Normal* (0, 2 days) | *Exponential* (rate 0.72) |
| Embryonic mortality | *Normal*(0, 10 log odds) | *Normal* (0, 2.5 log odds) | – |
| Hatch weight | *Normal*(3, 5.64 mg) | *Normal* (0, 1.41 mg) | *Exponential* (rate 0.56) |
| Hatch standard length | *Normal*(5, 3.21 mm) | *Normal* (0, 0.8 mm) | *Exponential* (rate 0.32) |
| Hatch yolk area | *Normal*(0, 2.5 mm^2^) | *Normal*(0, 0.63 mm^2^) | *Exponential* (rate 0.25) |

The prior distributions are provided in italics and the prior means and standard deviations in brackets unless otherwise specified. Majority of the standard deviations were acquired by multiplying the standard deviation of the dependent variable by 10 via the *rstanarm* package.

**Table S2.** Summary statistics.

| *Model*  Treatment | *n* | Median | 50% CI | 95% CI | Probability treatment < control | Probability treatment > control |
| --- | --- | --- | --- | --- | --- | --- |
| *Breeding probability* |  |  |  |  |  |  |
| 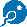 | 19 pairs | 34% | 19-41% | 5-67% | – | – |
| 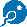 | 17 pairs | 58% | 46-72% | 23-91% | 15% | 85% |
| 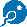 | 17 pairs | 26% | 12-33% | 2-60% | 64% | 36% |
| 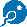 | 10 pairs | 38% | 20-50% | 4-79% | 44% | 56% |
| 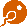 | 19 pairs | 30% | 17-38% | 3-64% | 57% | 43% |
| 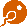 | 11 pairs | 3% | <0.01-3 | <0.01-22% | 98% | 2% |
| 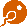 | 17 pairs | 19% | 6-22% | 1-50% | 79% | 21% |
| 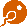 | 13 pairs | <0.01% | <0.01-<0.01% | <0.01-0.01% | 99.98% | 0.02% |
| *Clutch size* (eggs) |  |  |  |  |  |  |
| 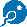 | 7 pairs | 327 | 297-349 | 247-407 | ­– | – |
| 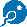 | 7 pairs | 297 | 273-320 | 222-370 | 76% | 24% |
| 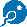 | 5 pairs | 308 | 278-333 | 224-404 | 65% | 35% |
| 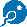 | 4 pairs | 240 | 217-265 | 168-316 | 96% | 4% |
| 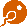 | 6 pairs | 323 | 295-350 | 239-418 | 53% | 47% |
| 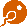 | 4 pairs | 308 | 274-336 | 223-423 | 62% | 38% |
| *Total eggs per pair* |  |  |  |  |  |  |
| 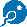 | 6 pairs | 681 | 567-744 | 424-995 | – | – |
| 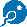 | 6 pairs | 654 | 543-720 | 395-992 | 57% | 43% |
| 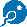 | 5 pairs | 436 | 355-479 | 247-636 | 96% | 4% |
| 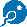 | 4 pairs | 457 | 358-496 | 267-710 | 94% | 6% |
| 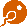 | 5 pairs | 539 | 442-594 | 333-810 | 84% | 16% |
| 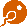 | 3 pairs | 359 | 263-391 | 176-594 | 97% | 3% |
| *Egg area* (mm^2^) |  |  |  |  |  |  |
| 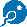 | 72 eggs | 4.25 | 4.11-4.38 | 3.82-4.64 | – | – |
| 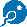 | 70 eggs | 4.48 | 4.35-4.62 | 4.05-4.94 | 17% | 83% |
| 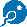 | 52 eggs | 4.15 | 3.99-4.30 | 3.67-4.60 | 67% | 33% |
| 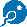 | 40 eggs | 4.35 | 4.19-4.53 | 3.83-4.85 | 36% | 64% |
| 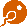 | 55 eggs | 4.11 | 3.96-4.25 | 3.68-4.55 | 72% | 28% |
| 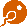 | 40 eggs | 3.76 | 3.60-3.94 | 3.24-4.29 | 96% | 4% |
| *Embryonic duration* (days) |  |  |  |  |  |  |
| 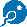 | 7 clutches | 9 | 9-9 | 9-10 | ­– | – |
| 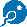 | 7 clutches | 9 | 9-9 | 9-10 | 57% | 43% |
| 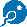 | 4 clutches | 10 | 9-10 | 9-10 | 11% | 89% |
| 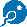 | 4 clutches | 10 | 10-10 | 9-10 | 4% | 96% |
| 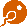 | 6 clutches | 8 | 8-8 | 8-9 | 99.8% | 0.2% |
| 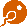 | 4 clutches | 8 | 8-8 | 8-9 | 99% | 1% |
| *Embryonic mortality* |  |  |  |  |  |  |
| 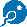 | 2224 eggs | 4% | 1-5% | 0.2-18% | – | – |
| 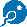 | 1811 eggs | 6% | 0.8-6% | 0.1-30% | 39% | 61% |
| 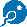 | 1513 eggs | 12% | 0.9-13% | 0.2-52% | 19% | 81% |
| 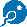 | 963 eggs | 4% | 0.2-5% | <0.01-27% | 48% | 52% |
| 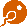 | 1487 eggs | 12% | 1-13% | 0.1-49% | 18% | 82% |
| 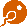 | 1193 eggs | 13% | 0.4-13% | 0.2-55% | 19% | 81% |
| *Hatch weight* (mg) |  |  |  |  |  |  |
| 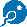 | 138 hatchlings | 3.3 | 3.2-3.4 | 3.0-3.6 | – | – |
| 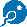 | 160 hatchlings | 3.5 | 3.3-3.6 | 2.9-3.9 | 26% | 74% |
| 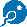 | 80 hatchlings | 3.4 | 3.2-3.6 | 2.8-3.9 | 37% | 63% |
| 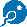 | 80 hatchlings | 3.4 | 3.3-3.7 | 2.8-4.0 | 30% | 70% |
| 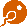 | 120 hatchlings | 3.0 | 2.6-3.0 | 2.6-3.5 | 82% | 18% |
| 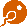 | 58 hatchlings | 2.8 | 2.9-3.2 | 2.3-3.4 | 93% | 6% |
| *Hatch standard length* (mm) |  |  |  |  |  |  |
| 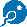 | 138 hatchlings | 5.16 | 5.08- 5.24 | 4.90-5.40 | – | – |
| 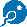 | 158 hatchlings | 5.14 | 5.05-5.22 | 4.88- 5.41 | 54% | 46% |
| 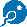 | 80 hatchlings | 5.03 | 4.93-5.12 | 4.72-5.32 | 83% | 17% |
| 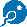 | 78 hatchlings | 5.09 | 5.00-5.20 | 4.77-5.37 | 69% | 31% |
| 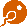 | 120 hatchlings | 4.91 | 4.82-5.00 | 4.62-5.17 | 97% | 3% |
| 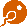 | 58 hatchlings | 5.06 | 4.94-5.15 | 4.74-5.37 | 76% | 24% |
| *Hatch yolk area* (mm^2^) |  |  |  |  |  |  |
| 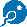 | 138 hatchlings | 1.47 | 1.44-1.49 | 1.38-1.56 | – | – |
| 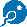 | 156 hatchlings | 1.69 | 1.64-1.73 | 1.53-1.84 | 0.7% | 99.3% |
| 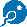 | 80 hatchlings | 1.54 | 1.49-1.60 | 1.37-1.71 | 21% | 79% |
| 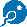 | 79 hatchlings | 1.71 | 1.65-1.76 | 1.53-1.88 | 1% | 99% |
| 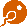 | 120 hatchlings | 1.44 | 1.38-1.48 | 1.28-1.58 | 67% | 33% |
| 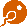 | 56 hatchlings | 1.56 | 1.49-1.62 | 1.37-1.76 | 17% | 83% |

Probabilities are expressed as a percent and the closer they are to 0% or 100% suggests greater confidence in a treatment being different relative to the control (
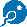
) whereas nearer to 50% suggests less confidence in a treatment being different relative to the control (
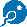
). Blue represents the present-day control temperature (in summer 28.5°C with ±0.6°C diurnal variation), orange represents a temperature increase of 1.5°C (in summer 30.0°C with ±0.6°C diurnal variation). The male and female symbols represent the developmental period and the egg and sperm icon represent the reproductive period. CI is Bayesian credible interval (analogous to a Frequentist confidence interval).

**Figure S1.** Bayesian posterior median values (circles), 50% credible intervals (rectangles), and 95% credible intervals (thin lines) of the A) embryonic duration, *n* = clutches and B) embryonic mortality, *n* = eggs. For logistical reasons (and as in the wild) offspring were kept with their parents until hatching. This meant embryos developed at their parents’ reproductive temperature. Blue represents the present-day control temperature (in summer 28.5°C with ±0.6°C diurnal variation), orange represents a temperature increase of 1.5°C (in summer 30.0°C with ±0.6°C diurnal variation).

**Literature cited**

Harrison, X. A. (2014). Using observation-level random effects to model overdispersion in count data in ecology and evolution. *PeerJ* doi:10.7717/peerj.616

Vehtari, A., Gelman, A., Gabry, J. (2017). Practical Bayesian model evaluation using leave-one-out cross-validation and WAIC. *Statistics and Computing* doi: 10.1007/s11222-016-9696-4
